# Effects of Weighted Vest Sprint Training on Mid-Acceleration and Reactive Strength in Post-PHV Soccer Players

**DOI:** 10.3390/sports14030124

**Published:** 2026-03-23

**Authors:** Nikola Stojanović, Branislav Majkić, Jadranka Vlašić, Valentin Barišić, Damir Pekas

**Affiliations:** 1 Faculty of Sport and Physical Education, University of Niš, 18000 Nis, Serbia; banesb@live.com; 2 Faculty of Kinesiology, University of Zagreb, 10000 Zagreb, Croatia; jadranka.vlasic@kif.unizg.hr (J.V.); valentin.barisic@kif.unizg.hr (V.B.); damir.pekas@kif.hr (D.P.)

**Keywords:** weighted vest sprint training, biological maturation, sprint acceleration, countermovement jump, youth soccer

## Abstract

Background: This study examined the effects of an individualized weighted vest sprint training program on sprint performance and countermovement jump (CMJ) outcomes in post-peak height velocity (PHV) male youth soccer players while accounting for maturation status. Methods: Fifty players (mean age 17.76 ± 0.95 years) were randomly assigned to a weighted vest sprint group (WVG; *n* = 25) or a traditional unloaded sprint group (TS; *n* = 25). Sprint performance (5, 10, 20, and 30 m) and CMJ-derived variables (jump height, peak power output, reactive strength index modified (RSI-modified), and eccentric rate of force development) were assessed before and after an 11-week intervention performed twice weekly, with the WVG completing sprint drills while wearing a weighted vest (~11% body mass). Results: Weighted vest sprint training produced greater improvements in 10 m sprint performance and RSI-modified (*d* = 1.37 and 1.55, respectively). However, after Benjamini–Hochberg adjustment for multiple comparisons, the effects were no longer statistically significant and should therefore be interpreted with caution. Maturity offset did not meaningfully moderate training-induced adaptations. Conclusions: These findings suggest that weighted vest sprint training may provide potential benefits for mid-acceleration performance and reactive strength in post-PHV youth soccer players, although the magnitude of these effects remains uncertain.

## 1. Introduction

Biological maturation is a major determinant of physical performance in youth soccer, strongly influencing sprint speed, muscular strength, power output, and neuromuscular coordination [1]. Peak height velocity (PHV), defined as the period of maximal growth in stature during adolescence, is commonly used as a practical indicator of maturation status and has been closely associated with changes in physical performance capacities [2,3]. In soccer, earlier-maturing athletes typically display greater body mass, muscle size, and strength, which often translate into temporary advantages in speed and power-based tasks [4]. Conversely, later-maturing players may rely more on technical proficiency and movement efficiency and may demonstrate favorable long-term development trajectories [5]. However, classifying athletes simply as pre- or post-PHV overlooks the substantial biological and neuromuscular variability that persists beyond PHV. Post-PHV athletes continue to undergo meaningful adaptations in muscle morphology, neural activation, and coordination that can influence sprinting and jumping performance [1,6]. Treating post-PHV players as a homogeneous group may therefore obscure important developmental differences relevant to training responsiveness.

Importantly, the timing and magnitude of these adaptations vary considerably between individuals, even among athletes classified as post-PHV [7]. Such variability suggests that athletes at different stages of post-maturational development may not respond uniformly to the same training stimulus. Contemporary models of youth athletic development emphasize that training effectiveness is closely linked to biological readiness rather than chronological age, highlighting the need for developmentally appropriate loading strategies [8,9]. Consequently, grouping all post-PHV athletes together may obscure meaningful differences in adaptation potential, potentially influencing how athletes respond to specific sprint training stimuli.

Sprint acceleration and maximal speed are decisive physical qualities in soccer, underpinning many high-intensity match actions such as breakaways, defensive recoveries, and transitional phases of play [10]. Match analysis studies consistently indicate that the majority of sprint efforts in soccer occur over relatively short distances, typically 5–20 m, and are frequently repeated throughout the match in response to rapidly changing tactical situations [11,12,13]. Moreover, these accelerative actions rarely occur in perfectly linear trajectories, as players often perform them while adjusting body orientation, responding to opponents, or transitioning between offensive and defensive phases of play. Consequently, the ability to rapidly generate acceleration over short distances and repeatedly reproduce these efforts under variable movement constraints represents a critical physical determinant of soccer performance. From a practical perspective, training interventions aimed at improving acceleration capacity must therefore be interpreted not only in terms of isolated sprint outcomes but also in relation to the functional demands encountered during match play. To enhance these qualities, resisted sprint training has been proposed as a sprint-specific method to increase mechanical loading during acceleration while preserving movement specificity. Systematic reviews and experimental studies, predominantly using sled towing, have demonstrated that appropriately prescribed resisted sprinting can improve sprint performance across short and longer distances, likely through enhanced horizontal force production and sprint-specific strength [14,15]. Although most research has focused on sled resistance, weighted vest sprinting offers an alternative means of applying external load while maintaining natural sprint mechanics and requiring minimal equipment. Experimental evidence in soccer players shows that sprint training with weighted vests can improve sprint and repeated-sprint performance [16]. Furthermore, applied training interventions incorporating weighted vests in adolescent soccer contexts have reported gains in sprint, change-of-direction, and jump performance [17]. Meta-analytic evidence also suggests that vest-resisted sprint training can produce meaningful improvements in linear sprint performance in young soccer players, although responses may vary depending on loading strategies and individual characteristics [18]. However, the effectiveness of resisted sprint modalities across different stages of post-PHV maturation remains insufficiently explored, particularly in adolescent soccer players.

While resisted sprint training can enhance sprint performance, the magnitude and quality of adaptation depend heavily on how training load and fatigue are managed. High-intensity sprint efforts place substantial neuromuscular and mechanical demands on athletes, and excessive fatigue can impair sprint mechanics and reduce force production, limiting the effectiveness of subsequent repetitions [14,19,20]. Traditional fixed-volume sprint training approaches fail to account for individual differences in fatigue tolerance and recovery capacity, which may be particularly pronounced in adolescent athletes undergoing ongoing biological development [8,19]. As a result, athletes may either accumulate excessive fatigue or fail to achieve sufficient high-quality sprint exposure to maximize adaptation. Performance-based dosing strategies based on performance decline, such as velocity or time loss thresholds, have been widely implemented as autoregulatory methods to individualize training volume in high-intensity resistance exercise to manage fatigue and preserve movement quality [21]. Comparable principles have been conceptually extended to sprint training, where declines in performance reflect accumulating fatigue and reductions in mechanical output [14,19]. In applied practice, small drop-off thresholds, commonly around 3%, are often implemented to preserve near-maximal sprint quality while limiting excessive fatigue accumulation. By ensuring that each sprint repetition is performed under optimal mechanical conditions, such approaches may enhance training efficiency and promote favorable neuromuscular adaptations, particularly when combined with high-demand resisted sprinting modalities.

Despite extensive research linking biological maturation to physical performance in youth soccer, relatively few studies have examined how variability within post-PHV athletes influences responsiveness to resisted sprint training, particularly when training volume is regulated using performance-based dosing strategies. Most investigations have treated post-PHV players as a relatively homogeneous population [1,2]. Consequently, the extent to which distinct stages of post-maturational development modify responses to resisted sprint training remains unclear. Moreover, although sprint training has been shown to confer performance benefits, most sprint interventions still prescribe fixed training volumes, and the use of performance-based dosing strategies that regulate sprint volume in response to performance decline has received relatively little empirical attention. Therefore, the present study aimed to investigate the effects of resisted sprint training combined with performance-based dosing on sprint performance and countermovement jump outcomes in post-PHV soccer players, while examining the moderating role of maturity offset. It was hypothesized that athletes undertaking resisted sprint training with performance-based dosing would demonstrate potentially greater improvements in sprint performance compared with traditional fixed-volume sprint training, and that the magnitude of these adaptations would vary according to maturity offset within the post-PHV period.

## 2. Materials and Methods

### 2.1. Study Design and Procedures

This longitudinal randomized study examined the influence of resisted sprint training combined with performance-based dosing across post-PHV maturation stages in male youth soccer players aged 16 to 19 years over an 11-week intervention period. Participants were randomly assigned to either a weighted vest training group (WVG; *n* = 25) or a traditional sprint group (TS; *n* = 25). The TS group served as an active comparison group, as both groups completed structured sprint training throughout the intervention period. The intervention comprised 22 sprint training sessions conducted twice weekly, during which the WVG group performed sprint drills while wearing a weighted vest corresponding to approximately 11% of individual body mass, whereas the TS group performed identical sprint drills without external load. All training sessions were scheduled at 17:00 h to ensure consistency of daily training timing and were separated by a minimum of 48 h. Sprint training was implemented following a standardized warm-up protocol, and participants continued their regular soccer training and match participation throughout the study period, including nine official and three friendly matches. All players followed the same team-based training program, which was supervised by the same coaching staff and identical for both groups throughout the intervention. Players also participated in the same competitive period, including official and friendly matches. However, individual match exposure (e.g., minutes played) was not controlled and may have varied between players. Performance assessments were conducted at baseline and after completion of the 11-week intervention under standardized conditions at 17:00 h to minimize potential circadian influences on neuromuscular performance. Testing was performed across two consecutive days. On the first day, anthropometric measurements, including standing height, sitting height, and leg length, were collected to estimate maturity offset relative to peak height velocity. On the second day, sprint performance over 5, 10, 20, and 30 m and countermovement jump (CMJ) performance were assessed following a standardized warm-up consisting of aerobic activity, dynamic mobility exercises, and progressive sprint and plyometric drills. A 30 min passive recovery period separated the sprint and CMJ testing to minimize fatigue effects. At least 90 s of rest were provided between consecutive sprint and jump trials. All testing was conducted on an artificial grass surface, and participants were instructed to refrain from high-intensity physical activity for 48 h prior to each testing session.

### 2.2. Sample Size Estimation

**Figure 1:**
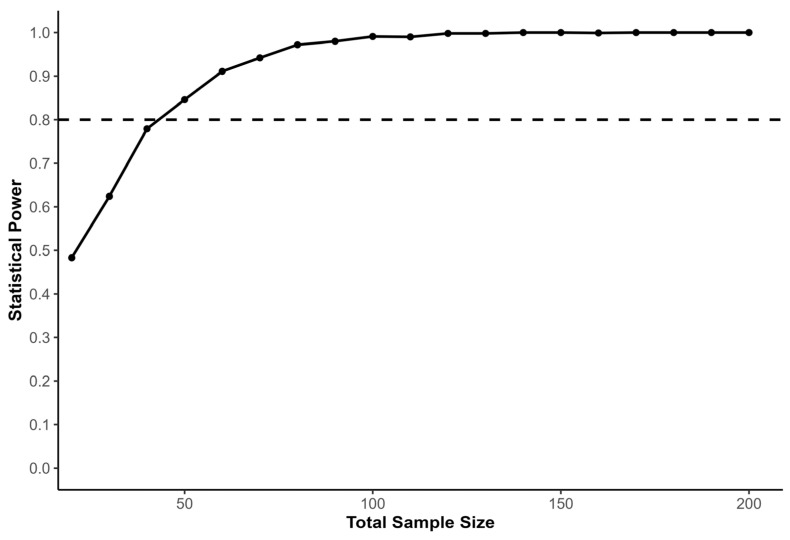
Power curve illustrating the estimated statistical power for detecting the group effect in a linear mixed-effects model including fixed effects for time, group, and their interaction, with participant entered as a random intercept. The x-axis represents the total sample size, while the y-axis shows the proportion of simulations in which the group effect was statistically significant (*p* < 0.05). The black dashed line marks the conventional 80% power threshold, indicating the minimum sample size required for sufficient statistical power. The curve shows that increasing the sample size improves the ability to detect the expected training effect while accounting for individual variability.

We conducted a Monte Carlo power analysis using simulated datasets to estimate the sample size required to detect a training effect within a linear mixed-effects modeling framework. Monte Carlo simulation was selected because it allows statistical power to be estimated under complex analytical structures that mirror the final statistical model used in the study. The primary outcome variable was 5 m sprint time, selected due to its sensitivity to acceleration-oriented performance adaptations, whereas longer sprint distances increasingly reflect maximal-speed mechanical characteristics [22,23]. Short sprint distances are used in research examining acceleration performance in team-sport athletes and are included as outcome variables in studies of resisted sprint training [15,22]. Baseline sprint performance was simulated to reflect observed variability in youth soccer players (*M* = 1.17 s, *SD* = 0.03), and pre-to-post performance changes were generated assuming a trivial mean effect in the TS (*d* = 0.10, *SD* = 0.09) and a moderate effect in the WVG (*d* = 0.55, *SD* = 0.15), consistent with reported adaptations following resisted sprint training interventions targeting acceleration performance [15]. The expected experimental-group effect size was set slightly below the value reported in the cited study (*d* = 0.65) in order to provide a conservative estimate of the anticipated training effect. Standardized effect sizes were converted to absolute sprint time changes using the baseline standard deviation, ensuring simulated performance changes reflected realistic sprint performance values. The simulated LME model included fixed effects for time, group, and their interaction, with participant identity entered as a random intercept to account for repeated measures. Within each simulation iteration, synthetic datasets were generated under the specified parameter assumptions, and the linear mixed-effects model was fitted via maximum likelihood to evaluate the statistical significance of the time × group interaction term. Power was estimated across total sample sizes ranging from 20 to 200 participants using 1000 simulation iterations per condition. Statistical power was defined as the proportion of simulations in which the interaction effect reached the predefined significance threshold (α = 0.05). The resulting power curve (Figure 1) illustrates the relationship between sample size and statistical power, with the conventional 80% threshold indicating the minimum required sample size. The analysis indicated that approximately 25 participants per group were required to achieve adequate power to detect the expected training effect.

### 2.3. Randomization and Blinding

This study employed block randomization with block sizes of 4 and 6 to ensure balanced allocation of 50 participants between the WVG and TS groups. Each participant was assigned a unique identification code linked to their group allocation, determined by sealed opaque envelopes containing the randomization assignments. Given the visible nature of the weighted vest intervention, blinding of participants and coaches was not feasible. However, test administrators were blinded to group allocation during both baseline and post-intervention assessments. The primary investigator remained blinded to group assignments until baseline testing was completed to reduce potential bias in study administration. A neutral administrator generated the randomization sequence, prepared and sealed the allocation envelopes, enrolled participants, and maintained allocation concealment until testing was completed. For statistical analysis, group assignments were coded to ensure objectivity.

### 2.4. Participants

The sample comprised 50 male soccer players aged 16 to 19 years competing at the highest national level. Participants, coaches, and club personnel were informed about the study procedures, experimental protocol, and research objectives prior to participation. Players were randomly assigned to either a weighted vest group (WVG; *n* = 25; age: 17.81 ± 0.94 years; body mass: 72.20 ± 6.56 kg; height: 178.90 ± 6.33 cm; body fat: 10.73 ± 3.81%; muscle mass: 49.90 ± 4.52%; maturity offset: 3.14 ± 0.78 years) or a traditional sprint training group (TS; *n* = 25; age: 17.71 ± 0.97 years; body mass: 74.70 ± 5.55 kg; height: 178.68 ± 3.97 cm; body fat: 10.47 ± 3.45%; muscle mass: 49.47 ± 5.08%; maturity offset: 3.09 ± 0.57 years). These maturity offset values indicate that all participants were several years beyond peak height velocity, confirming that despite the range in chronological age (16–19 years), the sample represented a biologically mature post-PHV cohort. Written informed consent was obtained from all participants, with parental or guardian consent provided for those under 18 years of age. Following baseline testing, all participants attended an instructional session outlining the training procedures. Inclusion criteria required that players were free from serious musculoskeletal injury within the preceding three months, attended at least 85% of all training sessions, and completed a minimum of 95% of the assigned sprint training sessions. The study was conducted in accordance with the Declaration of Helsinki and received approval from the Ethics Committee of the Faculty of Sport and Physical Education, University of Niš, Serbia (Decision No: 04-474/3; approval date: 28 March 2024). All randomized participants completed the intervention and post-testing procedures (*n* = 25 per group), and no dropouts or training-related injuries occurred during the study period. The intervention was conducted during the competitive phase of the season, during which player availability and participation remained stable.

### 2.5. Anthropometric Characteristics and Maturity Assessment

Anthropometric measurements were conducted using a Martin anthropometer (GPM 101, GPM GmbH, Susten, Switzerland) according to standardized procedures. Standing height, sitting height, and leg length were measured with participants positioned upright to ensure measurement accuracy. Body composition variables, including body fat percentage and muscle mass, were assessed using a bioelectrical impedance analyzer (InBody 770, InBody Co., Ltd., Seoul, Republic of Korea) following the manufacturer’s guidelines. Biological maturation was estimated using anthropometric variables: standing height (cm), sitting height (cm), leg length (cm), body mass (kg), and chronological age, with birth and testing dates recorded for each participant. Maturity status was expressed as maturity offset, representing the estimated number of years from peak height velocity (PHV), as described by Mirwald et al. [3]. For male participants, maturity offset was calculated using the following equation:

$$\begin{array}{cc} Maturity\;Offset\; & \left(years\right)\\ & =-9.236+\left(0.0002708\times Leg\;Length\times Sitting\;Height\right)\\ & -\left(0.001663\times Age\times Leg\;Length\right)+\left(0.007216\times Age\times Sitting\;Height\right)\\ & +\left(0.02292\times \frac{Body\;Mass}{Standing\;Height}\right) \end{array}$$

### 2.6. Experimental Program and Load Determination

**Figure 2:**
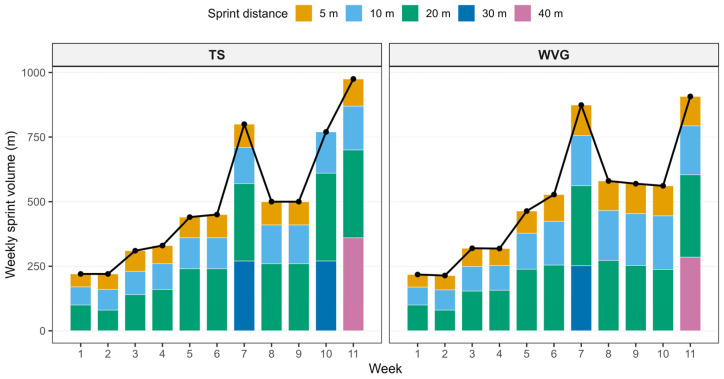
Comparison of weekly sprint training volume between the weighted vest group (WVG) and the traditional sprint group (TS) across the 11-week intervention period. Bars represent the mean weekly sprint distance for each group at each sprint distance (5 m, 10 m, 20 m, 30 m, and 40 m), with colors indicating the sprint distance. The black line with black points represents the mean total weekly sprint training distance for each group. Panels display the two groups separately to illustrate the progression and distribution of sprint training volume throughout the intervention period.

Each training session commenced with a standardized 12 min warm-up comprising aerobic activity (5 min), dynamic mobility exercises (5 min), and potentiation drills (2 min), designed to optimize sprint performance and reduce injury risk [19]. The aerobic component consisted of progressive low-intensity running, including jogging, lateral shuffles, and backward running, performed at approximately 50–60% of perceived maximal effort. The dynamic mobility phase included locomotor mobility drills targeting the hip, knee, and ankle joints, such as walking lunges with trunk rotation, straight leg marches, and dynamic hip openers performed over short distances at a controlled, moderate tempo. The final potentiation phase consisted of sprint mechanics drills (Sprint ABC), including A-skips, high-knee runs, and fast ankle dribbles, each performed once over approximately 10 m with progressively increasing movement frequency. The drills were executed sequentially with brief active recovery while walking back to the starting position before initiating the next drill. The sequence concluded with two progressive accelerations over 10–15 m, reaching near-maximal intensity, with walking recovery between runs, to increase neuromuscular readiness for subsequent maximal sprint efforts. Sprint training was performed immediately following the warm-up, while all participants maintained their regular team-based training routines throughout the intervention period, which were consistent across groups. The 11-week sprint training program followed a periodized structure [24]. During the initial four weeks, emphasis was placed on sprint technique development across distances of 5, 10, and 20 m, with progressive increases in training volume in subsequent phases. Both groups completed identical sprint distances and standardized recovery intervals. Rest between repetitions was set at 15 s, with a 1 min recovery following short sprint efforts and a 3 min recovery following longer sprint efforts [25,26]. Planned performance peaks were implemented in weeks 7 and 11 (Figure 2). In the weighted vest group (WVG), sprint sets were terminated when sprint time exceeded the athlete’s best performance within the session by more than 3% for two consecutive repetitions, thereby applying a performance-based dosing strategy to regulate training volume and preserve sprint quality. For each sprint distance, the fastest time achieved during the session served as the reference value for subsequent repetitions. This reference was updated if a faster time was recorded, but was not reset between sets performed at the same distance. Consequently, once the best time for a given distance was established, all subsequent repetitions were compared against this value. If the sprint time exceeded this reference by more than 3% for two consecutive repetitions, the set was terminated. The same procedure was applied independently for each sprint distance (e.g., 5 m, 10 m, 20 m). In contrast, the traditional sprint group (TS) performed the same sprint distances without external load and completed the prescribed number of repetitions irrespective of performance decrement. Across the intervention period, the WVG accumulated a slightly greater total sprint distance (*M* = 5553 m) compared with the TS group (*M* = 5115 m), corresponding to an approximate 8.6% difference in overall volume, indicating broadly comparable sprint exposure between groups. However, the distribution of volume across sprint distances differed as an inherent consequence of the autoregulatory approach (see Figure 2). Specifically, WVG accumulated greater total volume at 5 m (1009 vs. 805 m; Δ = +204 m) and 10 m (1633 vs. 1350 m; Δ = +283 m), but lower volume at 20 m (2374 vs. 2460 m; Δ = −86 m), 30 m (252 vs. 540 m; Δ = −288 m), and 40 m (285 vs. 360 m; Δ = −75 m). This structured variation may reflect individualized fatigue tolerance rather than uncontrolled training imbalance and was intentionally embedded within the performance-based dosing strategy. The sprint distance was selected as a practical indicator of training volume, given the standardized sprint distances and recovery structure across sessions. While sprint distance served as the primary volume metric, the weighted vest group performed all sprint efforts under an additional external load equivalent to approximately 11% of individual body mass, thereby increasing mechanical demand per repetition. Sprint performance during training sessions was monitored using photocell timing gates (Witty, Microgate, Bolzano, Italy). Real-time performance feedback was provided via a custom-developed application that recorded and graphically displayed sprint times across repetitions, enabling accurate implementation of the drop-off threshold.

The external load was determined individually using the equation:

$$\%\;\mathrm{body}\;\mathrm{mass}=-2.0762\times {\%\;V}_{\max}+207.99$$

where V_max_ represented each participant’s peak sprint velocity, and external resistance was individualized according to a velocity-based weighted vest loading approach as described by Carlos-Vivas et al. [27]. Use of this individualized velocity-based prescription resulted in an average external load of approximately 11% of body mass across participants. All athletes completed the prescribed sprint sessions without compliance issues, and no adverse responses to the weighted vest loads were reported during the intervention period. Arguably, this method may allow training volume to be prescribed relative to sprint performance capacity, thereby increasing mechanical demand while preserving sprint-specific movement patterns. Maximum sprint velocity was assessed using a Stalker ATS II radar system (Applied Concepts, Richardson, TX, USA), positioned five meters behind the starting line to ensure unobstructed measurement. Participants performed three maximal 30 m sprints with three-minute recovery intervals to minimize fatigue, and the fastest trial was used to determine individual peak velocity and prescribe the corresponding external load.

### 2.7. Sprint Test (30 m with Split Times at 5, 10, 20, and 30 m)

Sprint performance was assessed using photocell timing gates (Witty, Microgate, Bolzano, Italy) to record split times at 5, 10, 20, and 30 m. Participants started from a high-start position with the front foot placed 30 cm behind the start line and performed self-initiated maximal sprints through the final gate without intentional deceleration [28]. Standardized verbal encouragement from testers and peers was provided to support maximal effort across trials [29]. Each participant completed three trials, and the fastest 30 m sprint was retained for performance analyses. Measurement reliability was evaluated using intraclass correlation coefficients derived from a linear mixed-effects model (ICC[3,1]) and complemented by within-subject coefficients of variation (CV) to quantify absolute measurement error. Reliability was good to excellent across sprint splits, with ICC[3,1] values of 0.87, 0.92, 0.95, and 0.98 for 5, 10, 20, and 30 m, respectively, and low within-subject CV values (0.92%, 0.86%, 0.52%, and 0.43%), indicating stable and precise sprint-time assessments.

### 2.8. Countermovement Jump (CMJ) Data Processing and Variable Calculation

Participants performed countermovement jumps (CMJs) on a high-precision force plate (Kistler QuattroJump 9290DD, Winterthur, Switzerland) with a sampling rate of 500 Hz. Each trial began from an upright position with hands on the hips, followed by a descent before executing a maximal vertical jump. Participants completed three trials with at least 30 s of rest between attempts and maintained a static position beforehand to record body weight (N) for calibration. Raw force-time data were processed in MATLAB R2021a (MathWorks, Natick, MA, USA) using a fourth-order Savitzky–Golay filter (window length = 15). The variable calculation method is provided in Table 1. Measurement reliability for countermovement jump variables was assessed using intraclass correlation coefficients obtained from a linear mixed-effects model and complemented by within-subject coefficients of variation. Reliability was good to excellent across all outcomes, with ICC[3,1] values of 0.95 for jump height, 0.96 for RSI, 0.91 for eccentric rate of force development, and 0.90 for peak power. Absolute reliability was also acceptable, with within-subject CV values of 2.82% for jump height, 4.26% for RSI, 11.5% for eccentric RFD, and 2.41% for peak power output, indicating stable and consistent measurement across trials.

### 2.9. Statistical Analyses

All statistical analyses were performed in R (version 4.2.0) using RStudio (2026.01.0+392). Descriptive statistics (means and standard deviations) were calculated for all sprint and CMJ outcomes by group (weighted vest vs. traditional sprint) and across post-PHV maturity offsets. Primary inferences were obtained using linear mixed-effects models fitted with the nlme package. For each outcome, models included fixed effects for time (baseline vs. final), group, centered maturity offset, and their interactions, with a random intercept for each participant to account for repeated measures. Training volume (centered) was included as a covariate with a time-by-volume interaction, and baseline CMJ depth was additionally controlled for in all CMJ models. Model assumptions were evaluated using residual diagnostics to assess normality and homoscedasticity. Model-based changes over time were estimated using marginal trends with the emmeans package, with the primary inferential parameter for each outcome being the time × group interaction, representing the between-group difference in change over time. To account for multiple testing across outcomes, the Benjamini–Hochberg correction was applied to the outcome-level time × group interaction *p*-values. In addition, continuous conditional effects across the full observed maturity range were generated to examine whether the estimated between-group difference in change varied with maturity offset. Model-implied changes (final minus baseline) were also estimated separately for each group in raw units at the mean maturity level and, for CMJ outcomes, at the mean baseline CMJ depth. Standardized effects were obtained by dividing these conditional differences by the model residual standard deviation, with 95% confidence intervals derived from the corresponding model-based estimates. Effect sizes were interpreted according to Hopkins’ thresholds as trivial (<0.20), small (0.20 to 0.59), moderate (0.60 to 1.19), large (1.20 to 1.99), and very large (≥2.00) [30]. In addition, a separate linear regression model was used to examine the association between accumulated training volume and sprint performance outcomes, with maturity offset included as a covariate. Statistical significance was set at *p* < 0.05, and all results are presented with 95% confidence intervals.

## 3. Results

Table 2 summarizes baseline and final values, together with relative percentage changes, for sprint and countermovement jump (CMJ) performance variables in the traditional sprint (TS) and weighted vest (WVG) groups. The descriptive statistics provide an overview of training-related trends prior to model-based inference, showing comparable baseline performance between groups and modest improvements across most outcomes, with generally larger relative gains observed in the WVG group, particularly for early sprint acceleration and reactive CMJ-derived measures. Standardized baseline differences were trivial to small for most outcomes (*g* ≤ 0.25), although moderate differences were observed for the 20 m and 30 m sprint times. Importantly, the subsequent longitudinal mixed-effects models estimated treatment effects from group differences in change over time, while accounting for individual baseline levels, thereby reducing the influence of baseline imbalance when evaluating intervention effects.

**Figure 3:**
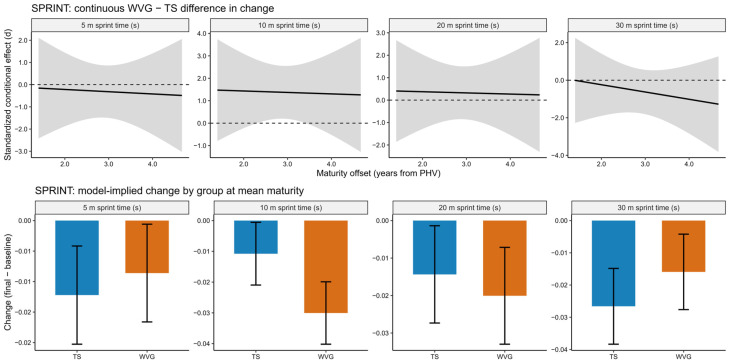
Model-based sprint performance outcomes across 5, 10, 20, and 30 m for the weighted vest (WVG) and traditional sprint (TS) groups in post-PHV athletes. The upper panels show the continuous standardized conditional effect representing the difference in change between WVG and TS across the observed maturity range, expressed as standardized effect sizes (*d*) with shaded bands indicating 95% confidence intervals. The dashed horizontal lines indicate the zero-effect reference line. The lower panels display model-implied changes (final minus baseline) in raw units for each group evaluated at the mean maturity level, with bars representing estimated changes and error bars indicating 95% confidence intervals. Positive values in the upper panels indicate a favorable effect of weighted vest training compared with traditional sprint training.

Model diagnostics indicated adequate fit across all outcomes, with residual distributions supporting normality and homoscedasticity and no evidence of problematic multicollinearity (see diagnostic plots). All participants were post-PHV adolescents (16 to 19 years), allowing the analysis to focus specifically on training adaptations within a biologically mature cohort. Sprint performance improved over time in both groups; however, the magnitude of change differed across sprint phases (Figure 3). For the 5 m sprint, between-group differences in change were trivial to small (*d* = −0.27 to −0.39) and not statistically significant (*p* = 0.677). Similarly, effects at 20 m remained small (*d* = 0.28 to 0.35; *p* = 0.589), while at 30 m differences favored the TS but were small to moderate (*d* = −0.44 to −0.90) and non-significant (*p* = 0.496), indicating no clear advantage of unweighted sprinting for maximal-speed phases. In contrast, a distinct benefit of weighted vest sprint training was observed in the 10 m sprint. The WVG demonstrated greater improvements than the TS, with a large standardized between-group difference in change (*d* = 1.37, 95% CI: 0.17 to 2.56; *p* = 0.026). However, after applying the Benjamini–Hochberg correction for multiple outcomes, the adjusted *p*-value exceeded the conventional significance threshold (BH-adjusted *p* = 0.104). The model-implied improvement ranged from approximately 0.019 to 0.020 s, particularly at lower and median maturity offsets. Training volume (total sprint distance) was included as a centered covariate with a time × training volume interaction and showed a moderate association with sprint performance, most notably in the 30 m sprint (*p* = 0.036), suggesting that higher accumulated volume might have contributed to sprint-speed adaptations. Importantly, adjusting for training volume did not alter the primary between-group findings, with the WVG maintaining a distinct advantage only for the 10 m sprint distance. No meaningful interactions involving maturity offset were observed across sprint outcomes, and the continuous conditional effect estimates remained relatively stable across the observed maturity range, indicating that biological maturity did not substantially moderate responsiveness to either training approach (see Figure 3).

**Figure 4:**
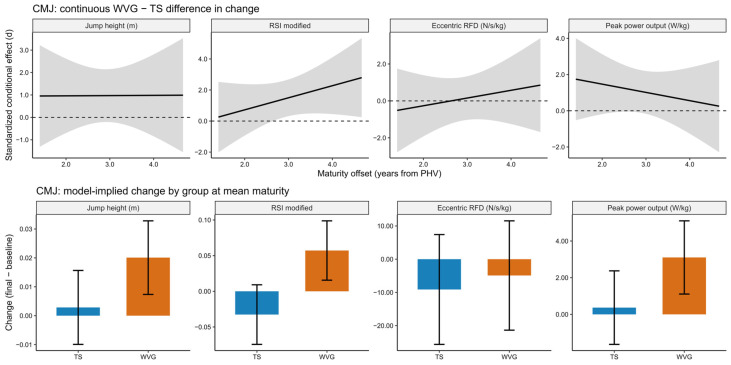
Model-based countermovement jump (CMJ) performance outcomes across jump height, RSI modified, eccentric RFD, and peak power output for the weighted vest (WVG) and traditional sprint (TS) groups in post-PHV athletes. The upper panels show the continuous standardized conditional effect representing the difference in change between WVG and TS across the maturity continuum, expressed as standardized effect sizes (*d*) with shaded bands indicating 95% confidence intervals. The dashed horizontal lines indicate the zero-effect reference line. The lower panels display model-implied changes (final minus baseline) in raw units for each group evaluated at the mean maturity level, with bars representing estimated changes and error bars indicating 95% confidence intervals. Positive values in the upper panels indicate a favorable effect of weighted vest training compared with traditional sprint training.

Model diagnostics likewise confirmed an appropriate fit for all CMJ outcomes, with residuals indicating normality and homoscedasticity, and no multicollinearity concerns. For jump height, differences in change between WVG and TS were moderate to large in magnitude but did not reach statistical significance across maturity offsets (Δ = 0.017 m; *d* = 0.97 to 0.98; *p* = 0.137), and no significant time × group interaction was observed (*p* = 0.108). Eccentric rate of force development (RFD) exhibited trivial to small effects in favor of WVG (*d* = −0.05 to 0.45), with wide confidence intervals and no statistically significant group differences (*p* = 0.936) or meaningful interaction effects (Figure 4). Peak power output showed moderate to large standardized differences favoring WVG (*d* = 0.70 to 1.24), with model-implied changes ranging from approximately 1.9 to 3.4 W/kg; however, these differences did not reach statistical significance (*p* = 0.058), and the time × group interaction remained non-significant (*p* = 0.104). In contrast, RSI-modified demonstrated a differential training response. The WVG improved substantially more than TS, with a large standardized between-group difference in change (*d* = 1.55, 95% CI: 0.36 to 2.75; *p* = 0.012). However, after applying the Benjamini–Hochberg correction for multiple outcomes, the adjusted *p*-value exceeded the conventional significance threshold (BH-adjusted *p* = 0.096). Additionally, a significant time × training volume interaction was observed for RSI-modified (*β* = −0.0002, *p* = 0.007), suggesting that training volume partially moderated the magnitude of RSI adaptation. No meaningful interactions involving maturity offset were observed across CMJ outcomes overall; however, the continuous conditional effect estimates suggested that the between-group advantage for RSI-modified tended to become more pronounced toward the higher end of the observed maturity range, although this pattern should be interpreted cautiously given the non-significant time × group × maturity interaction and the widening uncertainty around the conditional estimates (see Figure 4).

To further explore the role of training volume, a regression model examining total accumulated load showed that 30 m sprint performance was significantly associated with accumulated training exposure, independent of biological maturity (*β* = −1461, 95% CI [−2419, −504]; standardized *β* = −0.56; *p* = 0.004). In contrast, maturity offset was not meaningfully associated with accumulated load (*p* = 0.345). The model explained approximately 40% of the variance in training volume (adjusted *R*^2^ = 0.35). These results suggest that faster athletes tended to tolerate or accumulate higher sprint volumes, and that training volume partially contributed to performance adaptations, particularly in reactive strength. However, accumulated volume alone did not explain the superior early-acceleration improvements observed in the weighted vest group.

## 4. Discussion

The present study examined whether an 11-week sprint intervention performed with an individualized weighted vest and a performance-based termination rule (3% sprint-time drop-off) produces different adaptations in sprint and CMJ outcomes than traditional unloaded sprint training in post-PHV male soccer players, and whether maturity offset moderates these responses. The main finding was a between-group advantage for the weighted vest group in 10 m sprint performance, reflected by a large standardized difference in change (*d* = 1.37). Although the unadjusted model-based test indicated a significant time × group interaction (*p* = 0.026), this effect did not remain statistically significant after controlling for multiple comparisons using the Benjamini–Hochberg procedure. Nevertheless, the magnitude of the estimated effect remained large, suggesting a potentially meaningful training advantage that should be interpreted cautiously given the associated uncertainty. In contrast, between-group differences for 5 m, 20 m, and 30 m sprint times were small to moderate and not statistically significant. Across CMJ outcomes, the most consistent differential adaptation was observed for RSI-modified, which showed a large standardized change. A similar pattern was observed for RSI-modified: the unadjusted model-based test indicated a significant time × group interaction (*p* = 0.012), but this effect likewise did not remain statistically significant after correction for multiple comparisons. Contrarily, jump height, peak power, and eccentric RFD showed effects that were directionally favorable in some cases but not statistically clear. However, the analyzed outcomes should not be viewed as a collection of unrelated endpoints, because the sprint variables were derived from the same sprint test, and the CMJ variables were derived from the same countermovement jump test. Applying a global false-discovery-rate correction across all these derived measures may impose a multiplicity framework broader than the underlying measurement structure and should therefore be interpreted cautiously. Accordingly, the present findings are interpreted with emphasis on the magnitude and precision of the estimated effects, while treating adjusted *p*-values as a sensitivity check rather than the sole basis for inference. Importantly, maturity offset did not meaningfully moderate the training response within this post-PHV sample, because maturity-related interactions were not significant across outcomes. Finally, the ancillary volume analysis indicated that better 30 m sprint performance was associated with greater accumulated sprint volume capacity in the weighted vest condition (maturity offset not associated), supporting the interpretation that training exposure is partly performance-linked rather than maturity-driven within this cohort.

Importantly, because both groups performed structured sprint training throughout the intervention period, the present design compares two sprint training modalities rather than evaluating sprint training against a passive control condition. The selective improvement observed in the 10 m sprint, with no clear between-group advantage at 5 m or at longer distances (20 m and 30 m), suggests that weighted vest sprint training may preferentially influence performance during the mid-acceleration phase. This pattern should not be interpreted as evidence of speed decay beyond 10 m. Rather, the model-implied changes indicate that both groups improved across sprint distances, whereas the weighted vest condition appeared to confer a potential additional advantage specifically at 10 m. This phase of sprinting is characterized by progressive changes in posture and step kinematics as athletes transition from the initial steps toward a more upright running position [31]. The lack of a measurable benefit at 5 m may indicate that the external loading strategy did not substantially alter the neuromuscular demands of the earliest acceleration steps, which are strongly dependent on rapid force production and technical execution [15,32]. This observation may be practically relevant in the context of soccer match play, where many decisive actions occur over very short distances, and rapid accelerations within the first few meters are often required to gain positional advantage in both offensive and defensive situations [11,12,13,33]. Similarly, the absence of clear improvements at 20 m and 30 m suggests that the intervention may not have meaningfully affected maximal velocity or late-acceleration mechanics, which have been shown to rely on distinct biomechanical determinants such as stride characteristics and elastic energy utilization [20,23]. Taken together, the present findings suggest that weighted vest sprint training may elicit phase-specific adaptations within the acceleration continuum, with the clearest effect observed during the transition from early to mid-acceleration rather than across the full sprint distance spectrum. These findings are broadly consistent with recent meta-analytic evidence indicating that resisted sprint modalities, including both weighted vest and sled-based approaches, generally produce moderate improvements in acceleration performance in youth soccer players, while effects on maximal sprint velocity are more variable and dependent on the specific loading strategy employed [18]. From an applied football perspective, this finding may still be meaningful. Match analyses consistently show that many high-intensity actions in soccer occur over short distances, typically within 5 to 20 m, and are repeated in tactically constrained situations requiring rapid positioning, pressing, recovery runs, and short explosive movements to create separation from an opponent [10,11,12,13,33]. Accordingly, an intervention that preferentially improves performance in the early-to-mid acceleration phase may have practical utility even in the absence of clear changes in maximal sprint velocity. In addition, although the present study did not directly assess sprint kinematics, resisted sprinting with a weighted vest may have influenced movement organization during repeated accelerative efforts by requiring athletes to maintain effective force application and coordination under increased inertial demands [34]. This interpretation remains speculative, but it offers a plausible link between the observed phase-specific adaptation and the movement demands encountered in soccer.

The phase-specific nature of the observed sprint adaptations may partly reflect the directional characteristics of the applied external load. Unlike sled towing, weighted-vest sprinting increases system mass while allowing free running without a backward towing vector, thereby modifying sprint mechanics under added inertial demand [34]. In contrast, resisted sprint modalities that impose horizontal force demands, such as sled towing, have been shown to more directly overload horizontal force production and are therefore more closely associated with improvements in early acceleration performance [15,32]. Within this context, the weighted vest may be viewed as a complementary resisted sprint method that is more likely to enhance short acceleration qualities than to induce broad improvements across the full sprint distance spectrum. Biomechanical analyses further indicate that sprint acceleration mechanics shift progressively as posture becomes more upright and step kinematics change with increasing velocity [31]. Within this context, the present findings are consistent with the principle of training specificity, which holds that neuromuscular adaptations tend to reflect the direction and nature of the imposed mechanical demands [35]. Accordingly, while weighted vest sprint training may have elicited meaningful improvements in 10 m sprint performance, the lack of clear benefits for the earliest acceleration phase may be related to the limited horizontal overload provided by vertical loading alone, although the present study did not directly assess sprint mechanics or ground reaction forces. Similar performance enhancements following a weighted vest sprint training program have been reported in male soccer players, with improvements observed primarily in short-distance sprint performance and repeated-sprint ability compared with unloaded sprint training [16]. Importantly, the significant time × volume interaction observed for 30 m sprint performance suggests that accumulated sprint exposure may have influenced changes in longer sprint outcomes during the intervention period. Consistent with this observation, a complementary regression analysis demonstrated that faster 30 m sprint performance was associated with accumulated training volume, suggesting that athletes with higher sprint capacity tended to tolerate or accumulate greater sprint volumes. Together, these findings might indicate that training volume was partially performance-linked and may have contributed to sprint adaptation, particularly in outcomes influenced by repeated high-quality sprint efforts. This performance-linked accumulation of training exposure aligns with previous work in elite youth soccer, highlighting substantial inter-individual variability in load tolerance and adaptive responses when using RPE-based training load monitoring and underscoring the importance of individualized workload regulation when targeting high-intensity neuromuscular qualities [36]. In addition, although not examined inferentially, the autoregulatory approach inherently altered the distribution of sprint exposure across distances, with the weighted vest group accumulating relatively greater exposure to shorter sprint efforts and comparatively less exposure to longer sprint efforts. From a mechanistic perspective, this structured pattern of exposure may have further contributed to adaptations specific to the mid-acceleration phase while limiting stimulus directed toward maximal velocity development. Nevertheless, because distance-specific volume differences were not modeled statistically, this interpretation should be considered a plausible explanatory framework rather than a direct causal finding.

Beyond sprint performance, the present findings indicate that weighted vest sprint training elicited selective neuromuscular adaptations in countermovement jump performance. While jump height demonstrated moderate to large improvements favoring the weighted vest group, these differences did not reach statistical significance, suggesting a positive but variable transfer effect from resisted sprinting to vertical explosive performance. In contrast, peak power output and eccentric rate of force development showed no clear between-group differences, indicating that the applied sprint-specific overload may not have provided a sufficient stimulus to induce broad adaptations across the force–velocity spectrum. These results align with the principle of movement and force specificity, whereby training-induced improvements tend to manifest most strongly in tasks sharing similar mechanical and neuromuscular demands [35]. Weighted vest sprinting may increase loading demands and muscular recruitment during acceleration, thereby improving concentric impulse and stretch-shortening cycle utilization relevant to jump height, while not sufficiently targeting maximal power production or rapid eccentric force generation. This selective transfer suggests that resisted sprint training alone may improve certain explosive qualities but is unlikely to develop all components of lower-limb power without complementary strength or plyometric stimuli. In line with this interpretation, recent evidence from resisted small-sided games interventions in youth soccer has demonstrated improvements in selected power-related outcomes without uniform enhancement across all neuromuscular performance measures, highlighting the task-specific nature of resistance-based training stimuli [17]. Meta-analytic evidence further indicates that more pronounced improvements in vertical jump height typically occur when plyometric or targeted power-based interventions are incorporated alongside sport-specific training, supporting the notion that resisted sprinting alone may not provide a sufficiently comprehensive stimulus for maximal jump performance development [37].

Although weighted vest sprint training did not produce statistically greater improvements in countermovement jump height than traditional sprint training, it was associated with substantially larger gains in the reactive strength index modified (RSI-modified), indicating a more pronounced enhancement in the efficiency of force production relative to movement time during the countermovement jump. RSI-modified is calculated as jump height divided by time to take-off and reflects an athlete’s capacity to rapidly generate impulse through coordinated eccentric braking and concentric propulsion within the CMJ movement [38,39]. Therefore, while jump height primarily reflects the magnitude of the concentric impulse achieved during takeoff, RSI-modified is more sensitive to the temporal characteristics of force application and the effectiveness of the eccentric-to-concentric transition. This is consistent with biomechanical analyses of countermovement jumping, which emphasize the critical role of eccentric braking and subsequent force reutilization in enhancing stretch-shortening cycle efficiency and impulse generation [40]. The greater improvements in RSI-modified observed in the weighted vest group suggest that resisted sprinting may preferentially enhance neuromuscular qualities related to efficient force transfer rather than maximal concentric output alone. We might argue that sprinting under additional vertical load likely increased braking demands during stance, requiring greater force absorption and redirection within short contact times. Repeated exposure to these elevated eccentric loading conditions may have promoted adaptations in neuromuscular coordination and force-time characteristics that favor faster impulse generation during the CMJ, thereby improving RSI-modified performance without necessarily producing proportionally greater jump height gains [35,41]. Importantly, the unadjusted significant group × time interaction for RSI-modified suggested that the two training approaches elicited distinct neuromuscular adaptations over the intervention period, confirming a clear treatment-related effect, although this effect did not remain statistically significant after correction for multiple comparisons. It should also be noted that the autoregulatory protocol slightly shifted sprint exposure toward shorter acceleration distances in the weighted vest condition, which may have contributed to the observed reactive strength adaptations. From a mechanistic perspective, greater repeated exposure to high-intensity efforts within shorter acceleration phases, combined with additional vertical loading, may have increased the frequency of eccentric braking and rapid force redirection, both of which are closely linked to stretch-shortening cycle efficiency. As such, while the primary evidence suggests a treatment-related adaptation associated with weighted vest sprint training, the pattern of sprint exposure across distances may also have contributed to the observed reactive strength adaptations by shaping the mechanical stimulus experienced throughout the intervention. Nevertheless, because distance-specific volume was not modeled inferentially, this interpretation should be viewed as a complementary explanatory framework rather than a direct causal finding.

Although biological maturation is known to strongly influence baseline sprint and neuromuscular performance in youth athletes, the present findings indicate that maturity offset did not meaningfully moderate training-induced adaptations in either sprint or CMJ outcomes within this post-PHV cohort. Previous studies have consistently reported that more mature adolescents demonstrate superior sprint speed, force production, and stretch-shortening cycle function due to increases in muscle mass, neuromuscular coordination, and mechanical efficiency [1,2,6,7]. However, once athletes reach biological maturity, developmental processes appear to exert a diminished influence on responsiveness to training stimuli. Longitudinal research examining strength and power adaptations across maturation stages similarly suggests that training responsiveness may be heightened during earlier developmental phases, with more uniform adaptation patterns emerging as athletes approach or surpass peak height velocity [42]. In support of this interpretation, the present analysis revealed that faster 30 m sprint performance was a significant predictor of accumulated training volume, whereas maturity offset was not meaningfully associated with exposure capacity. This suggests that training tolerance and, possibly, subsequent adaptation were more closely linked to an athlete’s existing performance capacity than to their maturational status. This perspective is supported by empirical evidence from elite youth soccer, indicating that the contributions of speed and power qualities to performance outcomes vary by maturational stage, with physical capacities becoming increasingly decisive in later adolescence [43]. Similar observations have been reported in youth development models, which propose that as athletes progress into late adolescence, training responsiveness becomes increasingly governed by mechanical loading characteristics and individual capacity to tolerate high-intensity stimuli rather than by ongoing biological growth [8,9]. Collectively, these findings indicate that within post-PHV populations, performance level may be a more relevant determinant of training exposure and adaptive potential than developmental stage itself, helping to explain the relatively uniform responses to weighted vest sprint training observed across maturity offsets in the present study.

However, several limitations should be acknowledged. Moreover, accumulated sprint distance represents only a simplified external volume metric and does not fully characterize the mechanical demands of sprint training. The mechanical stimulus generated during sprinting depends not only on the distance covered but also on factors such as external resistance and the athlete’s force–velocity capabilities, which determine the force and velocity conditions under which each step is produced. Consequently, similar sprint distances may not necessarily reflect equivalent neuromuscular or mechanical demands across training conditions. The regression analysis linking sprint performance to accumulated load provides insight into exposure capacity but remains observational. The study was limited to post-PHV male soccer players, which restricts generalizability to younger athletes, female populations, and other sporting contexts. Biological maturation was estimated using the Mirwald maturity-offset equation [3], which is widely used in youth sport research. However, prediction error has been shown to increase when estimates are made further from the timing of peak height velocity [44,45,46]. Because the participants in the present study were, on average, approximately 3 years post-PHV, variability in maturity offset was relatively small, and the estimates should therefore be interpreted cautiously. In addition, the intervention duration may not have been sufficient to elicit adaptations in maximal velocity mechanics or broader strength-related neuromuscular qualities. Furthermore, direct kinematic and kinetic sprint variables were not assessed, limiting the ability to confirm the specific mechanical mechanisms underlying the observed phase-specific performance adaptations. In addition, because the autoregulatory design produced slight between-group differences in accumulated sprint exposure, the observed adaptations cannot be attributed solely to external loading independent of exposure-related influences. Finally, although the team-based training program was standardized across groups, individual match exposure during the competitive season (e.g., minutes played) was not controlled and may have contributed to variability in accumulated external load. Nonetheless, key strengths of the present study include the use of an active comparison group performing traditional sprint training, together with individualized weighted vest loading combined with a performance-based drop-off rule, allowing training stimuli to be tailored to each athlete’s capacity. The continuous modeling of maturity offset provided a refined assessment of developmental influences beyond categorical groupings, while the mixed-model framework enabled evaluation of treatment effects alongside exposure-related factors. The inclusion of multiple sprint distances and comprehensive CMJ-derived variables further allowed detailed examination of phase-specific sprint adaptations and selective transfer effects. An additional strength lies in applying the drop-off strategy within a sport-specific acceleration context, as regulating sprint volume based on performance decline may help preserve high-quality efforts over short acceleration distances, particularly relevant to soccer performance, while potentially fostering greater fatigue tolerance in this critical phase of sprinting. Future research should investigate individualized resisted sprint training across a broader range of maturational stages to determine whether performance capacity similarly governs adaptation in earlier developmental phases. Experimental manipulation of loading direction and distance-specific sprint volume would help clarify dose–response relationships and phase-specific mechanisms. Additionally, integrating resisted sprinting with complementary strength or plyometric interventions may promote more comprehensive neuromuscular adaptations. Longer-term longitudinal studies with refined workload modeling are also warranted to better understand how mechanical overload and training tolerance interact to shape sprint development.

## 5. Conclusions

The purpose of this study was to examine whether sprint training with an individualized weighted vest, combined with a performance-based drop-off rule, produces different adaptations in sprint and countermovement jump outcomes compared with traditional unloaded sprint training in post-PHV male soccer players, and whether maturity offset moderates these responses. This study demonstrated that sprint training performed with an individualized weighted vest and a performance-based drop-off rule elicited selective improvements in 10 m sprint performance and reactive strength (RSI-modified) compared with traditional unloaded sprint training in post-PHV male soccer players. Although the Benjamini–Hochberg adjustment yielded non-significant *p*-values, the estimated effects remained large and were accompanied by relatively wide confidence intervals, suggesting caution in interpretation. In contrast, between-group differences for 5 m, 20 m, and 30 m sprint times, as well as for CMJ height, peak power, and eccentric rate of force development, were small to moderate and not statistically significant. Maturity offset did not meaningfully moderate training-induced adaptations across sprint or CMJ outcomes, indicating relatively uniform responsiveness within biologically mature soccer players. Ancillary volume analyses further suggested that greater sprint performance capacity was associated with higher accumulated training exposure, supporting the role of performance-linked workload tolerance in shaping adaptation. Although training volume was included as a covariate, the weighted vest group accumulated slightly greater sprint volume due to the autoregulatory training design; therefore, the observed adaptations cannot be attributed solely to the external loading stimulus and may also reflect differences in training exposure. Collectively, these findings indicate that weighted vest sprint training may preferentially enhance mid-acceleration performance and reactive neuromuscular qualities, more consistent with adaptation likely influenced by mechanical loading and exposure capacity rather than developmental stage in post-PHV athletes. Nonetheless, maturity offset was estimated using an anthropometric prediction model and should therefore be interpreted with caution in athletes several years beyond PHV.

## Figures and Tables

**Table 1:** Calculation Method for Performance Variables.

Variable (Formula)
$JH=\frac{V_{\mathrm{takeoff}}^{2}}{2\times 9.81}$
$RSI_{\mathrm{mod}}=\frac{JH}{t_{\mathrm{takeoff}}-t_{1}}$
$RFD_{\mathrm{ecc}}=\frac{F_{z\mathrm{braking}\;\mathrm{end}}-F_{z\mathrm{unload}\;\mathrm{end}}}{t_{\mathrm{braking}\;\mathrm{end}}-t_{\mathrm{unload}\;\mathrm{end}}}\times \frac{1}{M}$
$P_{\mathrm{peak}}=\frac{P_{peak}}{M}$

Note: Calculation formulas for key performance variables. Jump height was derived from takeoff velocity. Peak power was the maximum instantaneous power output normalized to body mass. Eccentric RFD was the force difference between the braking and unloading phases, divided by the time interval and normalized to body mass. The modified reactive strength index was computed as jump height divided by the time from movement initiation to takeoff.

**Table 2:** Descriptive performance characteristics of participants in the traditional sprint (TS) and weighted vest (WVG) groups at baseline and post-intervention.

Outcome	Group	Baseline (*M* ± *SD*)	Final (*M* ± *SD*)	Delta (%)
5 m sprint time (s)	TS	1.180 ± 0.032	1.170 ± 0.030	−0.8%
	WVG	1.172 ± 0.028	1.161 ± 0.032	−1.0%
10 m sprint time (s)	TS	1.944 ± 0.053	1.932 ± 0.052	−0.6%
	WVG	1.941 ± 0.053	1.912 ± 0.050	−1.5%
20 m sprint time (s)	TS	3.228 ± 0.075	3.214 ± 0.077	−0.4%
	WVG	3.166 ± 0.063	3.145 ± 0.067	−0.7%
30 m sprint time (s)	TS	4.435 ± 0.108	4.416 ± 0.111	−0.4%
	WVG	4.347 ± 0.117	4.323 ± 0.123	−0.5%
Jump height (m)	TS	0.322 ± 0.049	0.326 ± 0.051	1.2%
	WVG	0.325 ± 0.056	0.344 ± 0.059	5.9%
RSI modified	TS	0.421 ± 0.123	0.424 ± 0.119	0.7%
	WVG	0.405 ± 0.100	0.427 ± 0.085	5.5%
Eccentric RFD (N/s/kg)	TS	69.781 ± 30.780	61.799 ± 26.191	−11.4%
	WVG	70.745 ± 50.355	64.868 ± 21.449	−8.3%
Peak power output (W/kg)	TS	49.163 ± 5.480	50.114 ± 6.682	1.9%
	WVG	49.684 ± 6.235	52.182 ± 6.867	5.0%

Note: Sprint times (5 m, 10 m, 20 m, 30 m), jump height, peak power output, reactive strength index (RSI modified), and eccentric RFD. Values are presented as mean ± standard deviation, with relative percentage change (Δ%) indicating the proportional difference from baseline to final assessment.

## Data Availability

The data presented in this study were collected specifically for this research and are not publicly available due to ethical and privacy considerations involving minor participants. Anonymized datasets and the R scripts used for statistical analyses are available from the corresponding author upon reasonable request.

## Abbreviations

The following abbreviations are used in this manuscript:

CMJ	Countermovement jump
RSI-mod	Reactive strength index modified
PHV	Peak height velocity
WVG	Weighted vest group
TS	Traditional sprint group
RFD	Rate of force development
SSC	Stretch-shortening cycle
